# A personalized approach for tumour biopsy in hepatocellular carcinoma

**DOI:** 10.1016/j.jhepr.2023.100979

**Published:** 2023-12-03

**Authors:** Maxime Ronot, Jean-Charles Nault

**Affiliations:** 1 Université de Paris, INSERM U1149 "Centre de Recherche sur l'inflammation", CRI, Paris, France; 2 Department of Radiology, AP-HP, Hôpital Beaujon APHP.Nord, Clichy, France; 3 Liver Unit, Hôpital Avicenne, Hôpitaux Universitaires Paris-Seine-Saint-Denis, Assistance-Publique Hôpitaux de Paris, Bobigny, France; 4 Unité de Formation et de Recherche Santé Médecine et Biologie Humaine, Université Paris 13, Communauté d’Universités et Etablissements Sorbonne Paris Cité, Paris, France; 5 Centre de Recherche des Cordeliers, Sorbonne Université, Inserm, Université de Paris, Team « Functional Genomics of Solid Tumors », F-75006 Paris, France; 6 Equipe labellisée Ligue Nationale Contre le Cancer, Labex OncoImmunology, France

To the Editor:

We read with great interest the study by Brusset *et al.* about the role of biopsy and imaging in diagnosing hepatocellular carcinoma (HCC).^1^ The authors implemented a policy of systematic biopsy of suspected HCC in their centre and share their initial experience. They show that a small subset of observations classified as LR-5 were not HCC, and some HCC subtypes (*e.g*., macrotrabecular massive), although rare, were often misclassified. The authors argue that their study emphasizes the limits of the imaging-based non-invasive diagnosis of HCC and supports the need for systematic biopsy when HCC is suspected. We work in institutions that enforce similar “systematic biopsy of suspected HCC” policies and strongly believe in the complementary role of pathology and imaging for managing liver malignancies. Therefore, we want to congratulate the authors for their work. However, their results raise several points that need clarification.

As a preliminary statement, we want to stress that a non-invasive diagnostic system such as the LI-RADS can only be objectively assessed if properly applied.[4], [5] The authors claim that they included only patients at high risk according to the LI-RADS, which is good, but they indicate that histologically F3 fibrosis was observed in 32 patients, which is not considered as high risk *per se*, except in patients with HBV or a history of HCC.^5^ Also, proper adherence to the system terminology (*e.g*., “arterial phase hyperenhancement” and not “hypervascularization at the arterial phase”, “ancillary features” and not “minor features”) is encouraged.

More importantly and clinically relevant is the actual oncological benefit one may anticipate from a systematic biopsy policy. This is of utmost importance if a shift from non-invasive to pathological diagnosis of HCC is to be encouraged. Currently, histo-prognostic factors derived from biopsy analysis and genomic analysis to identify druggable genetic alterations are not implemented in clinical practice, and a systematic tumour biopsy is not recommended in the European guidelines.^2^ Nevertheless, we recognize that prognostic markers may hold potential for future applications in stratifying adjuvant trials. A recent pilot study has suggested that molecular-based targeted therapy is feasible for a subset of patients with advanced HCC.^3^ Additionally, tumour sampling is essential for translational research, a domain that remains less advanced in HCC compared to other types of solid cancers.

Overall, the decision on whether to biopsy all suspected HCC needs to consider the following points. First, not all HCCs are equally easily targetable. Some are located in challenging areas of the liver, others may be difficult to differentiate from a nodular liver background, the patient may not be compliant, *etc.* Second, even if a lesion is sampled, a subset of biopsies is not contributive. It was the case in 18% of biopsies in the study by Brusset *et al.*, which is consistent with published literature. Third, the current LI-RADS suggests biopsies should be performed (or at least discussed) in non-LR-5 observations (*e.g*., LR-4, LR-M and even LR-TIV). Altogether, the performance of different diagnostic strategies in an *“intention-to-diagnose”* perspective should be considered prospectively in terms of accuracy, rate of false negatives, and type of error. Indeed, comparing imaging to contributive biopsies can only be seen as a *“per-protocol”* analysis, with an optimistic bias toward biopsy. Even if the study by Brusset *et al.* is retrospective, could the authors provide these results based on their data and experience, especially comparing the “systematic biopsy” and the “LR-5 or biopsy” policies? We would also be interested in their opinion on what to do in case of a non-targetable lesion or non-contributive biopsy if a systematic biopsy policy is applied.

Finally, one major argument in favour of systematic biopsy raised by the authors is the possible influence on treatment decisions, but 63 patients (38%) had their biopsy during a thermal ablation procedure.^1^ In light of the results of their study, have the authors decided to change their practice and separate biopsies from ablations?

Once again, we thank the authors for their stimulating study that will help refine future recommendations.

## Financial support

The authors did not receive any financial support to produce this manuscript.

## Conflict of interests

The authors of this study declare that they do not have any conflict of interest.

Please refer to the accompanying ICMJE disclosure forms for further details.

## Authors' contributions

MR and JCN: drafting and final editing of the manuscript.
