# Supplementary material for: A personalized approach for tumour biopsy in hepatocellular carcinoma
Source: JHEP Rep. 2023 Dec 3;6(4):100979. doi: 10.1016/j.jhepr.2023.100979 (PMC10951645; doi:10.1016/j.jhepr.2023.100979)
Supplement: Multimedia component 2 [file mmc2.pdf]

A personalized approach for tumour biopsy in hepatocellular carcinoma

To the Editor:

We read with great interest the study by Brusset *et al.* about the role of biopsy and imaging in diagnosing hepatocellular carcinoma (HCC).[1] The authors implemented a policy of systematic biopsy of suspected HCC in their centre and share their initial experience. They show that a small subset of observations classified as LR-5 were not HCC, and some HCC subtypes (*e.g.*, macrotrabecular massive), although rare, were often misclassified. The authors argue that their study emphasizes the limits of the imaging-based non-invasive diagnosis of HCC and supports the need for systematic biopsy when HCC is suspected. We work in institutions that enforce similar "systematic biopsy of suspected HCC" policies and strongly believe in the complementary role of pathology and imaging for managing liver malignancies. Therefore, we want to congratulate the authors for their work. However, their results raise several points that need clarification.

As a preliminary statement, we want to stress that a non-invasive diagnostic system such as the LI-RADS can only be objectively assessed if properly applied.[4,5] The authors claim that they included only patients at high risk according to the LI-RADS, which is good, but they indicate that histologically F3 fibrosis was observed in 32 patients, which is not considered as high risk *per se*, except in patients with HBV or a history of HCC.[5] Also, proper adherence to the system terminology (*e.g.*, "arterial phase hyperenhancement" and not "hypervascularization at the arterial phase", "ancillary features" and not "minor features") is encouraged.

More importantly and clinically relevant is the actual oncological benefit one may anticipate from a systematic biopsy policy. This is of utmost importance if a shift from non-invasive to pathological diagnosis of HCC is to be encouraged. Currently, histo-prognostic factors derived from biopsy analysis and genomic analysis to identify druggable genetic alterations are not implemented in clinical practice, and a systematic tumour biopsy is not recommended in the European guidelines.[2] Nevertheless, we recognize that prognostic markers may hold potential for future applications in stratifying adjuvant trials. A recent pilot study has suggested that molecular-based targeted therapy is feasible for a subset of patients with advanced HCC.3 Additionally, tumour sampling is essential for translational research, a domain that remains less advanced in HCC compared to other types of solid cancers.

Overall, the decision on whether to biopsy all suspected HCC needs to consider the following points. First, not all HCCs are equally easily targetable. Some are located in challenging areas of the liver, others may be difficult to differentiate from a nodular liver background, the patient may not be compliant, *etc.* Second, even if a lesion is sampled, a subset of biopsies is not contributive. It was the case in 18% of biopsies in the study by Brusset *et al.*, which is consistent with published literature. Third, the current LI-RADS suggests biopsies should be performed (or at least discussed) in non-LR-5 observations (*e.g.*, LR-4, LR-M and even LR-TIV). Altogether, the performance of different diagnostic strategies in an *"intention-to-diagnose"* perspective should be considered prospectively in terms of accuracy, rate of false negatives, and type of error. Indeed, comparing imaging to contributive biopsies can only be seen as a *"per-protocol"* analysis, with an optimistic bias toward biopsy. Even if the study by Brusset *et al.* is retrospective, could the authors provide these results based on their data and experience, especially comparing the "systematic biopsy" and the "LR-5 or biopsy" policies? We would also be interested in their opinion on what to do in case of a non-targetable lesion or non-contributive biopsy if a systematic biopsy policy is applied.

Finally, one major argument in favour of systematic biopsy raised by the authors is the possible influence on treatment decisions, but 63 patients (38%) had their biopsy during a thermal ablation procedure.[1] In light of the results of their study, have the authors decided to change their practice and separate biopsies from ablations?

Once again, we thank the authors for their stimulating study that will help refine future recommendations.

Financial support

The authors did not receive any financial support to produce this manuscript.

Conflict of interests

The authors of this study declare that they do not have any conflict of interest.

Please refer to the accompanying ICMJE disclosure forms for further details.

Received 6 November 2023; accepted 6 November 2023; available online 3 December 2023

Authors' contributions

MR and JCN: drafting and final editing of the manuscript.

Supplementary data

Supplementary data to this article can be found online at https://doi.org/10.1016/j.jhepr.2023.100979.

References

[1] https://doi.org/10.1016/j.jhepr.2023.100957.

[2] European Association for the Study of the Liver. Electronic address eee, European Association for the Study of the L. EASL clinical practice guidelines: management of hepatocellular carcinoma. J Hepatol 2018;69: 182–236.

[3] Limousin W, Laurent-Puig P, Ziol M, et al. Molecular-based targeted therapies in patients with hepatocellular carcinoma and hepato-

ELSEVIER

EASL The Home of Hepatology

cholangiocarcinoma refractory to atezolizumab/bevacizumab. J Hepatol 2023;28(23):S0168–S8278. 05070-5.
[4] Cannella R, Dioguardi Burgio M, Sartoris R, et al. Adherence to LI-RADS and EASL high-risk population criteria: a systematic review. Hepatology 2023 Jun 1;77(6):1958–1967.
[5] CT/MRI LI-RADS® v2018 CORE [Internet]. Available from: https://www.acr.org/-/media/ACR/Files/RADS/LI-RADS/LI-RADS-2018-Core.pdf.

Maxime Ronot[1,2,*]
Jean-Charles Nault[3,4,5,6]

[1]*Université de Paris, INSERM U1149 "Centre de Recherche sur l'inflammation", CRI, Paris, France;*
[2]*Department of Radiology, AP-HP, Hôpital Beaujon APHP.Nord, Clichy, France;*
[3]*Liver Unit, Hôpital Avicenne, Hôpitaux Universitaires Paris-Seine-Saint-Denis, Assistance-Publique Hôpitaux de Paris, Bobigny, France;*
[4]*Unité de Formation et de Recherche Santé Médecine et Biologie Humaine, Université Paris 13, Communauté d'Universités et Etablissements Sorbonne Paris Cité, Paris, France;*
[5]*Centre de Recherche des Cordeliers, Sorbonne Université, Inserm, Université de Paris, Team « Functional Genomics of Solid Tumors » F-75006 Paris, France;*
[6]*Equipe labellisée Ligue Nationale Contre le Cancer, Labex OncoImmunology, France*

* Corresponding author. Address: Service de radiologie, Hôpital Beaujon, APHP.Nord 100, Bd du général Leclerc 92110, Clichy, France; Tel.: +33 1 4087 5566.
E-mail address: maxime.ronot@aphp.fr (M. Ronot).

Journal of Hepatology, Volume *6*

Supplemental information

A personalized approach for tumour biopsy in hepatocellular carcinoma

Maxime Ronot and Jean-Charles Nault

ICMJE DISCLOSURE FORM

Date: 11/28/2023

Your Name: Jean-Charles Nault

Manuscript Title: A personalized approach for tumour biopsy in hepatocellular carcinoma

Manuscript Number (if known): JHEPR-D-23-01150

In the interest of transparency, we ask you to disclose all relationships/activities/interests listed below that are related to the content of your manuscript. "Related" means any relation with for-profit or not-for-profit third parties whose interests may be affected by the content of the manuscript. Disclosure represents a commitment to transparency and does not necessarily indicate a bias. If you are in doubt about whether to list a relationship/activity/interest, it is preferable that you do so.

The author's relationships/activities/interests should be defined broadly. For example, if your manuscript pertains to the epidemiology of hypertension, you should declare all relationships with manufacturers of antihypertensive medication, even if that medication is not mentioned in the manuscript.

In item #1 below, report all support for the work reported in this manuscript without time limit. For all other items, the time frame for disclosure is the past 36 months.

		Name all entities with whom you have this relationship or indicate none (add rows as needed)	Specifications/Comments (e.g., if payments were made to you or to your institution)
Time frame: Since the initial planning of the work			
1	All support for the present manuscript (e.g., funding, provision of study materials, medical writing, article processing charges, etc.) **No time limit for this item.**	☒ None	
			Click the tab key to add additional rows.
Time frame: past 36 months			
2	Grants or contracts from any entity (if not indicated in item #1 above).	☐ **None**	
		Ipsen, Bayer	
3	Royalties or licenses	☒ **None**	

		Name all entities with whom you have this relationship or indicate none (add rows as needed)	Specifications/Comments (e.g., if payments were made to you or to your institution)
**4**	Consulting fees	☒ **None**	
**5**	Payment or honoraria for lectures, presentations, speakers bureaus, manuscript writing or educational events	☒ **None**	
**6**	Payment for expert testimony	☒ **None**	
**7**	Support for attending meetings and/or travel	☒ **None**	
**8**	Patents planned, issued or pending	☒ **None**	
**9**	Participation on a Data Safety Monitoring Board or Advisory Board	☒ **None**	
**10**	Leadership or fiduciary role in other board, society, committee or advocacy group, paid or unpaid	☒ **None**	

		Name all entities with whom you have this relationship or indicate none (add rows as needed)	Specifications/Comments (e.g., if payments were made to you or to your institution)
**11**	Stock or stock options	☒ **None**	
**12**	Receipt of equipment, materials, drugs, medical writing, gifts or other services	☒ **None**	
**13**	Other financial or non-financial interests	☒ **None**	

Please place an "X" next to the following statement to indicate your agreement:

☒ I certify that I have answered every question and have not altered the wording of any of the questions on this form.

ICMJE DISCLOSURE FORM

Date: 11/28/2023

Your Name: Maxime Ronot

Manuscript Title: A personalized approach for tumour biopsy in hepatocellular carcinoma

Manuscript Number (if known): JHEPR-D-23-01150

In the interest of transparency, we ask you to disclose all relationships/activities/interests listed below that are related to the content of your manuscript. "Related" means any relation with for-profit or not-for-profit third parties whose interests may be affected by the content of the manuscript. Disclosure represents a commitment to transparency and does not necessarily indicate a bias. If you are in doubt about whether to list a relationship/activity/interest, it is preferable that you do so.

The author's relationships/activities/interests should be defined broadly. For example, if your manuscript pertains to the epidemiology of hypertension, you should declare all relationships with manufacturers of antihypertensive medication, even if that medication is not mentioned in the manuscript.

In item #1 below, report all support for the work reported in this manuscript without time limit. For all other items, the time frame for disclosure is the past 36 months.

		Name all entities with whom you have this relationship or indicate none (add rows as needed)	Specifications/Comments (e.g., if payments were made to you or to your institution)
**Time frame: Since the initial planning of the work**			
1	All support for the present manuscript (e.g., funding, provision of study materials, medical writing, article processing charges, etc.) **No time limit for this item.**	☒ None	
			Click the tab key to add additional rows.
**Time frame: past 36 months**			
**2**	Grants or contracts from any entity (if not indicated in item #1 above).	☒ **None**	
**3**	Royalties or licenses	☒ **None**	

		Name all entities with whom you have this relationship or indicate none (add rows as needed)	Specifications/Comments (e.g., if payments were made to you or to your institution)
**4**	Consulting fees	☐ **None**	
		Quantum surgical	To institution
**5**	Payment or honoraria for lectures, presentations, speakers bureaus, manuscript writing or educational events	☒ **None**	
		General Electrics, Guerbet, Terumo, Servier, Angiodynamics, Astrazeneca	
**6**	Payment for expert testimony	☒ **None**	
**7**	Support for attending meetings and/or travel	☒ **None**	
**8**	Patents planned, issued or pending	☒ **None**	
**9**	Participation on a Data Safety Monitoring Board or Advisory Board	☒ **None**	
**10**	Leadership or fiduciary role in other board, society, committee or advocacy group, paid or unpaid	☒ **None**	

		Name all entities with whom you have this relationship or indicate none (add rows as needed)	Specifications/Comments (e.g., if payments were made to you or to your institution)
**11**	Stock or stock options	☒ **None**	
**12**	Receipt of equipment, materials, drugs, medical writing, gifts or other services	☒ **None**	
**13**	Other financial or non-financial interests	☒ **None**	

Please place an "X" next to the following statement to indicate your agreement:

☒ I certify that I have answered every question and have not altered the wording of any of the questions on this form.
